# Perceived stress and post-traumatic stress disorder symptoms among intensive care unit staff caring for severely ill coronavirus disease 2019 patients during the pandemic: a national study

**DOI:** 10.1186/s12991-021-00363-1

**Published:** 2021-08-21

**Authors:** Nisha Kader, Bushra Elhusein, Nirvana Swamy Kudlur Chandrappa, Abdulqadir J. Nashwan, Prem Chandra, Abdul Waheed Khan, Majid Alabdulla

**Affiliations:** 1 Mental Health Service, Hamad Medical Corporation, 3050 Doha, Qatar; 2 Hazm Mebaireek General Hospital, Hamad Medical Corporation, Doha, Qatar; 3 Medical Research Centre, Hamad Medical Corporation, Doha, Qatar; 4 College of Medicine, Qatar University, Doha, Qatar

**Keywords:** COVID-19, ICU, Nurses, Perceived stress, PTSD

## Abstract

**Background:**

Intensive care unit (ICU) staff have faced unprecedented challenges during the coronavirus disease 2019 (COVID-19) pandemic, which could significantly affect their mental health and well-being. The present study aimed to investigate perceived stress and post-traumatic stress disorder (PTSD) symptoms reported by ICU staff working directly with COVID-19 patients.

**Methods:**

The Perceived Stress Scale was used to assess perceived stress, the PTSD Diagnostic Scale for the Diagnostic and Statistical Manual of Mental Disorders (5th edition) was used to determine PTSD symptoms, and a sociodemographic questionnaire was used to record different sociodemographic variables.

**Results:**

Altogether, 124 participants (57.2% of whom were men) were included in the analysis. The majority of participants perceived working in the ICU with COVID-19 patients as moderately to severely stressful. Moreover, 71.4% of doctors and 74.4% of nurses experienced moderate-to-severe perceived stress. The staff with previous ICU experience were less likely to have a probable diagnosis of PTSD than those without previous ICU experience.

**Conclusions:**

Assessing perceived stress levels and PTSD among ICU staff may enhance our understanding of COVID-19-induced mental health challenges. Specific strategies to enhance ICU staff’s mental well-being during the COVID-19 pandemic should be employed and monitored regularly. Interventions aimed at alleviating sources of anxiety in a high-stress environment may reduce the likelihood of developing PTSD.

## Background

In light of several recent studies, there is evidence that the coronavirus disease 2019 (COVID-19) pandemic has caused several mental health concerns in the general population, as well as healthcare workers (HCWs) [1–3]. One study conducted during the COVID-19 pandemic revealed that, among HCWs in China, 50.3%, 44.6%, and 34% experienced depression, anxiety, and insomnia, respectively [4]. Another recent study conducted in Italy found that 49.4%, 24.7%, 19.8%, 8.3%, and 21.9% of HCWs reported post-traumatic stress disorder (PTSD) symptoms, severe depression, anxiety, insomnia, and high perceived stress, respectively [5].

Intensive care unit (ICU) staff have witnessed the unpredictable course of COVID-19, rapid deterioration of patients’ health, infections among colleagues due to the higher rates of transmission, and long-standing contact and exposure with infected patients daily [6]. These factors may render them a vulnerable population to perceive more stress and develop PTSD symptoms.

Stress can have a long-term impact on health; nonetheless, there is little consensus on which types and aspects of stress are most important for human health and disease. This is partly due to the fact that stress is an emergent process involving interactions between individual and environmental factors, historical and current events, allostatic states, and psychological and physiological reactivities [7]. The effect of stress on health has been documented in population-based studies that measured perception or exposure to stressors. Stress is inextricably associated with psychological well-being, and stressful events are a major contributor to many common mental disorders [8, 9].

At one point, PTSD was strongly linked to war veterans. Later, researchers and clinicians recognized that PTSD symptoms are widespread among individuals exposed to a stressful event or situation of an exceptionally threatening nature [10]. The American Psychiatric Association revised the PTSD diagnostic criteria in the 5th edition of the Diagnostic and Statistical Manual of Mental Disorders (DSM-5). PTSD is included under a new category in the DSM-5 titled Trauma- and Stress-Related Disorders [11].

Studies have demonstrated that occupational stress generally causes burnout and can lead to a lack of job satisfaction and negative attitudes toward work [12]. Factors that make the ICU a stressful place of employment include the increased complexity and demands of most job descriptions, increased number of ICU admissions, unpredictable changes in one’s daily work routine, unrealistic expectations from patients and their families, and frequent encounters with ethical and end-of-life issues [13–16].

Understanding the impact of COVID-19 on healthcare staff, particularly ICU staff, is critical [17]. There is growing evidence that ICU staff and health care workers in general are at risk of developing PTSD after being exposed to COVID-19 patients [18–20]. Mental health considerations in ICU staff are being increasingly recognized during this pandemic [21, 22]. Williamson et al. highlighted “moral injury” resulting from ICU experiences in HCWs [23]. Moreover, an increased incidence of anxiety and stress disorders was identified in a study of 230 medical staff managing COVID-19 patients [24].

The presence of perceived stress, PTSD, and PTSD symptoms in ICU staff in Qatar has not previously been studied. Screening ICU workers is critical as they are at the forefront of fighting the COVID-19 pandemic. Understanding and managing their mental health and psychological well-being during this time is just as important as understanding and managing their physical health.

## Methods

### Participants

The present cross-sectional survey was conducted between August 2020 and September 2020, after the peak of the first wave of the COVID-19 pandemic in Qatar. Our study aimed to identify the degree of perceived stress of working in the ICU during the pandemic and to determine the prevalence of PTSD or PTSD symptoms in ICU doctors and nurses working in direct contact with COVID-19 patients at Hazm Mebaireek General Hospital (HMGH). Emails containing the survey link were sent to all eligible participants through a group email. Data were collected via an anonymous self-reported questionnaire.

HMGH joined the network of Hamad Medical Corporation (HMC; primary healthcare provider in Qatar) in 2018, offering inpatient and outpatient care, surgical services, and intensive and emergency care. Before the pandemic, HMGH had only 16 ICU beds for both surgical and medical critically ill patients. In March 2020, HMGH was designated as the main COVID-19 treatment center for adult patients in Qatar. During the first wave of COVID-19, HMGH ICU’s bed capacity increased to 226 beds to provide the best possible care to patients with severe COVID-19 symptoms.

### Ethical statement

The authors assert that all procedures contributing to this work complied with the ethical standards of the relevant national and institutional committees on human experimentation and with the 1975 Declaration of Helsinki, as revised in 2008. All procedures involving human subjects were approved by HMC (Approval Number: MRC-05-047).

### Informed consent

Participation in the present study was voluntary, and anonymity was assured. All participants were informed that their agreement to take part in the survey would constitute their consent to participate.

### Measures

#### Perceived stress scale

The Perceived Stress Scale (PSS) is a classic stress assessment [25]. We used the Perceived Stress Scale (PSS) to determine perceived stress among ICU staff during the COVID-19 pandemic. We asked the participants to answer the questions in the context of working in ICU during COVID-19. Individual scores range from 0 to 40, with higher scores indicating higher perceived stress. Scores from 0 to 13, 14 to 26, and 27 to 40 are considered low, moderate, and high levels of perceived stress, respectively [26].

#### PTSD diagnostic scale for DSM-5

The PTSD Diagnostic Scale for DSM-5 (PDS-5) is a self-report measure of DSM-5 PTSD criteria based on the Posttraumatic Stress Scale—Interview [27]. Participants are instructed to rate the frequency and distress associated with each of the PTSD symptoms on a scale ranging from 0 (not at all) to 4 (six or more times a week, indicating severe pathology). The PDS-5 yields both a PTSD diagnosis according to the DSM-5 criteria and a measure of PTSD severity. PTSD severity is determined by summing the 20 symptom ratings (items 1–20). Scores range from 0 to 80, with the following clinical guidelines for PTSD symptom severity: 0–10 = minimal symptoms, 11–23 = mild symptoms, 24–42 = moderate symptoms, 43–59 = severe symptoms, 60–80 = very severe symptoms. The PDS-5 has been found to have excellent internal consistency for total symptom severity (Cronbach’s alpha = 0.94). Test–retest reliability for PDS is high (Cronbach’s alpha = 0.88). The PDS has also demonstrated high concurrent validity with the PTSD Checklist for DSM-5 (*r* = 0.85, *p* < 0.01) [28].

According to the PDS-5, a score of 28 was used as a cut off for the possible diagnosis of PTSD, with scores between 0 and 27 indicating no diagnosis, and 28–80 indicating probable diagnosis [28].

#### Sociodemographic questionnaire

A sociodemographic questionnaire was used to collect different sociodemographic variables, as shown in Table 1. This information was gathered to contextualize the data and examine sociodemographic correlates of stress and PTSD.

**Table 1:** Sociodemographic characteristics of the staff working in the intensive care unit (ICU) during the 2019 coronavirus pandemic

Sociodemographic variables	*n* (%)
Age	
20–34 years	88 (71.0)
35–64 years	36 (29.0)
Gender	
Male	71(57.2)
Female	53 (42.8)
Family status	
Single	45 (36.3)
Married	79 (63.7)
Occupation	
Doctor	7 (5.6)
Nurse	117 (94.4)
Number of years working in HMC	
< 1 year	19 (15.3)
1–3 years	66 (53.2)
4–6 years	18 (14.5)
> 6 years	21 (17.0)
Previous ICU experience	
Yes	99 (79.8)
No	25 (20.2)
Posted to ICU due to COVID-19	
Yes	103 (83.1)
No	21 (16.9)
Previous mental health issues	
Yes	5 (4.0)
No	119 (96.0)
Family history of mental illness	
Yes	9 (7.3)
No	115 (92.7)
Secondary stressors such as financial issues	
Yes	58 (46.8)
No	66 (53.2)

*n* = 124

### Statistical analysis

Quantitative and categorical data are presented as mean ± standard deviation and frequencies (percentages). Associations between two or more qualitative variables were assessed using the Chi-squared (*χ*^2^) or Fisher’s exact tests, as appropriate. Univariate and multivariate logistic regression analyses (adjusted for potential predictors and confounders) were performed to determine and assess the predictive value of potential predictors and risk factors (demographic characteristics and parameters related to clinical and other characteristics) associated with outcome variables of perceived stress and probable diagnosis of PTSD. The results of the logistic regression analyses are presented using odds ratio (OR) with corresponding 95% confidence intervals (CIs). Graphic presentations of key results were created using appropriate statistical graphs. All *p* values presented were two-tailed, and *p* < 0.05 was considered statistically significant. All statistical analyses were performed using SPSS (version 27.0; IBM, Armonk, NY, USA) and Epi Info (Centers for Disease Control and Prevention, Atlanta, GA, USA).

## Results

A total of 124 out of 143 participants returned the questionnaires with a response rate of 86.7%. More than two-thirds of the participants belonged to the 25–34-year age group, and there was a slight male preponderance (57.2% versus 42.8%).

The majority of the respondents were between the ages of 20 and 34 years [*n* = 88 (71.0%)] and married [*n* = 79 (63.7%)]. Most of the respondents were nurses [*n* = 117 (94.4%)], and 79.8% (*n* = 99) had previous ICU experience. The majority of participants [*n* = 103 (83.1%)] were assigned to the ICU due to the COVID-19 pandemic. Only 4.0% (*n* = 5) of participants had previous mental health concerns, and 7.3% (*n* = 9) had a family history of mental illness. Nearly half of the respondents [*n* = 58 (46.8%)] endorsed secondary stressors, such as financial problems. The multivariate logistic regression of respondents who had secondary stressors, such as financial problems, were found to remain significantly associated with a probable diagnosis of PTSD (adjusted OR 3.56; 95% CI 1.18–10.73; *p* = 0.024).

The sociodemographic characteristics of the respondents are presented in Table 1.

### Perceived stress among ICU staff

Although 25.8% (*n* = 32) perceived working in the ICU with COVID-19 patients as a low-stress activity, 67.7% (*n* = 84) and 6.5% (*n* = 8), perceived it as moderate and high stress activity, respectively. Moderate and severe perceived stress responses were grouped together (74.2%; *n* = 92) to assess their possible association with sociodemographic variables.

### Logistic regression of sociodemographic variables and moderate-to-high perceived stress

Three-quarters of the respondents [*n* = 92 (74.2%)] perceived working in the ICU during the COVID-19 pandemic as moderate-to-high-stress activity. A total of 78.4% (*n* = 69) of the 92 respondents in the 20‒34-year age group experienced moderate-to-severe psychological distress, and this proportion was 63.9% (*n* = 23) among respondents in the 35‒64-year age group (*p* = 0.093). Overall, 70.4% (*n* = 50) of men and 79.2% (*n* = 42) of women experienced moderate-to-severe perceived stress (*p* = 0.267). Approximately 80.0% of the respondents (*n* = 36) who were single and 70.9% (*n* = 56) who were married experienced moderate-to-severe perceived stress (*p* = 0.265).

Seven doctors and 117 nurses participated in the study. 71.4% (*n* = 5) of doctors and 74.4% (*n* = 87) of nurses experienced moderate-to-severe perceived stress (*p* = 0.863).

79.8% (99) of the respondents had previous ICU experience, and 20.2% (25) did not have previous ICU experience. Eighty percent (*n* = 20) of staff without previous ICU experience and 72.7% (*n* = 72) of staff with previous ICU experience reported moderate-to-high perceived stress (*p* = 0.458).

83.1% (103) of the respondents were newly assigned to ICU due to the COVID-19 pandemic compared to 16.9% (21) of the respondents already working in the ICU. Three-quarters of the respondents that were newly assigned to the ICU due to the COVID-19 pandemic [*n* = 78 (75.7%)] and 66.7% of staff who were already working in the ICU (*n* = 14) experienced moderate-to-high perceived stress (*p* = 0.387).

All staff with a history of mental health concerns (*n* = 5) reported moderate-to-severe stress when working in the ICU, while such levels of stress were reported by 73.1% (*n* = 87) of those who did not have a history of mental health difficulties (*p* = 0.178). Moreover, 77.8% (*n* = 7) of respondents with a family history of mental illness reported moderate-to-severe perceived stress, while such high levels were reported by 73.9% (*n* = 85) of those without a family history of these illnesses (*p* = 0.799). Furthermore, 86.2% (*n* = 50) of respondents who had secondary stressors, such as financial problems, experienced moderate-to-high stress levels, and this proportion was only 63.6% (*n* = 42) among those who did not have any secondary stressors (*p* = 0.004).

Univariate logistic regression was performed to assess the effect of sociodemographic variables and other clinical characteristics on the outcome variable of the moderate-to-high perceived stress group, as presented in Table 2. In the multivariate logistic regression, age and secondary stressors, such as financial problems, were found to remain significantly associated with moderate-to-severe perceived stress. Multivariate logistic regression indicated that respondents in the 20–34-year age group were more likely to experience moderate-to-severe perceived stress compared to the 35–64-year age group (adjusted OR 3.72; 95% CI 1.10–12.60; *p* = 0.035). Moreover, respondents who had secondary stressors, such as financial difficulties, were more likely to experience moderate-to-severe perceived stress compared with those who did not have these stressors (adjusted OR 4.49; 95% CI 1.67–12.12; *p* = 0.003).

**Table 2:** Effect of sociodemographic variables on moderate-to-high perceived stress

Sociodemographic variables	*n* (%)	OR (95% CI)	*p* value
Age			
20–34 years	69 (78.4)	2.05 (0.88–4.80)	0.097
35–64 years	23 (63.9)	1	
Gender			
Male	50 (70.4)	1	0.269
Female	42 (79.2)	1.60 (0.69–3.70)	
Family status			
Single	36 (80.0)	1.64 (0.68–3.95)	0.267
Married	56 (70.9)	1	
Occupation			
Doctor	5 (71.4)	0.86 (0.16–4.68)	0.863
Nurse	87 (74.4)	1	
Number of years working in HMC			
< 1 year	15 (78.9)	1.17(0.26–5.21)	0.835
1–3 years	48 (72.7)	0.83 (0.27–2.61)	0.754
4–6 years	13 (72.2)	0.81 (0.19–3.43)	0.777
> 6 years	16 (76.2)	1	
Previous ICU experience			
Yes	72 (72.7)	0.67 (0.23–1.95)	0.460
No	20 (80.0)	1	
Posted to ICU due to COVID-19			
Yes	78 (75.7)	1.56 (0.57–4.30)	0.390
No	14 (66.7)	1	
Family history of mental illness			
Yes	7 (77.8)	1.24 (0.24–6.28)	0.799
No	85 (73.9)	1	
Financial problems			
Yes	50 (86.2)	3.57 (1.45–8.78)	0.006
No	42 (63.6)	1	

Those who scored more than 14 on Perceived Stress Scale had moderate-to-high perceived stress; *n* = 92

*OR* Odds ratio, *CI* confidence interval

Although not statistically significant, variables such as previous ICU experience and assignment to the ICU due to COVID-19 were associated with moderate-to-high perceived stress. Respondents who were newly posted to the ICU were more likely to experience moderate-to-high stress compared with those who were already working in the ICU (adjusted OR 1.41; 95% CI 0.41–4.83; *p* = 0.589). Moreover, the staff with previous ICU experience were less likely to experience moderate-to-high stress than those without previous ICU experience (adjusted OR 0.46; 95% CI 0.13–1.70; *p* = 0.243).

#### PTSD symptoms among ICU staff

One hundred and two respondents (82.3%) scored less than 28, indicating no diagnosis; however, 17.7% (*n* = 22) scored more than 28, indicating a probable diagnosis of PTSD.

### Logistic regression of sociodemographic variables and probable diagnosis of PTSD

A total of 17.0% (*n* = 15) and 19.4% (*n* = 7) of respondents in the 20‒34-year and 35‒64-year age groups, respectively, had a result indicative of probable PTSD diagnosis (*p* = 0.751).

Overall, 16.9% (*n* = 12) of men and 18.9% (*n* = 10) of women had probable diagnosis of PTSD (*p* = 0.777). Approximately 13.3% of the respondents (*n* = 6) who were single and 20.3% (*n* = 16) who were married had a probable diagnosis of PTSD (*p* = 0.332). Finally, 28.6% (*n* = 2) of doctors and 17.1% (*n* = 20) of nurses had a probable diagnosis of PTSD (*p* = 0.440).

Furthermore, 24.0% (*n* = 6) of staff without previous ICU experience had a probable diagnosis of PTSD, and the proportion was 16.2% (*n* = 16) among staff with ICU experience (*p* = 0.359). Nineteen percent (*n* = 4) of respondents who were recently relocated to the ICU due to the COVID-19 pandemic and 17.5% (*n* = 18) of those who were already working in the ICU had a probable diagnosis of PTSD (*p* = 0.864).

Finally, 20.0% of staff with previous mental health difficulties (*n* = 1) and 17.6% (*n* = 21) of those who did not have a history of mental health issues had a probable diagnosis of PTSD (*p* = 0.893). Moreover, 25.9% (*n* = 15) of respondents who had secondary stressors, such as financial problems experienced moderate-to-high stress, and this proportion was only 10.6% (*n* = 7) among those who did not have any secondary stressors (*p* = 0.026). Univariate logistic regression of sociodemographic variables and patients with probable diagnosis of PTSD is presented in Table 3.

**Table 3:** Effect of sociodemographic variables on the probable diagnosis of post-traumatic stress disorder

Sociodemographic variables	*n* (%)	OR (95% CI)	*p* value
Age			
20–34 years	15 (17.0)	0.85 (0.32–2.30)	0.751
35–64 years	7 (19.4)	1	
Gender			
Male	12 (16.9)	1	
Female	10 (18.9)	1.14 (0.45–2.89)	0.777
Family status			
Single	6 (13.3)	0.61 (0.22–1.68)	0.335
Married	16 (20.3)	1	
Occupation			
Doctor	2 (28.6)	1.94 (0.35–10.72)	0.447
Nurse	20 (17.1)	1	
Number of years working in HMC			
< 1 year	1 (5.3)	0.24 (0.03–2.33)	0.217
1–3 years	14 (21.2)	1.14 (0.33–3.95)	0.831
4–6 years	3 (16.7)	0.85 (0.16–4.43)	0.847
> 6 years	4 (19.0)	1	
Previous ICU experience			
Yes	16 (16.2)	0.61 (0.21–1.77)	0.363
No	6 (24.0)	1	
Posted to ICU due to COVID-19			
Yes	18 (17.5)	0.90 (0.27–2.99)	0.864
No	4 (19.0)	1	
Previous mental health issues			
Yes	1 (20.0)	1.17 (0.12–10.97)	0.893
No	21 (17.6)	1	
Family history of mental illness			
Yes	1 (11.1)	0.56 (0.07–4.71)	0.593
No	21 (18.3)	1	
Secondary stressors such as financial issues			
Yes	15 (25.9)	2.94 (1.10–7.83)	0.031
No	7 (10.6)	1	

Those who scored more than 28 on PTSD Diagnostic Scale for DSM-5 (PDS-5) had probable diagnosis of PTSD; *n* = 22

*OR* Odds ratio, *CI* confidence interval

### Logistic regression of perceived stress and probable diagnosis of PTSD

Logistic regression indicated that respondents who scored moderate to high on the PSS were more likely to have a probable diagnosis of PTSD compared with those who scored low (OR 12.83; 95% CI 1.69‒97.36; *p* = 0.014). This association was more notable in participants who perceived working in the ICU during the COVID-19 pandemic as a high-stress activity than among those who perceived it as a source of low to moderate stress (OR 26.28; 95% CI 5.22‒132.30; *p* < 0.0001).

## Discussion

During the current COVID-19 pandemic, ICU staff may be at a heightened risk of psychological distress. In the present study, we focused on perceived stress and PTSD symptoms to better understand the needs of patients. Furthermore, it may represent a basis for developing stronger policies to sustain ICU staff through this challenging time.

ICU staff are confronted with unprecedented scenarios that frequently exceed their normal levels of experience and training, as they are at the forefront of the global fight against the virus. The severity increases the risk of ICU staff experiencing symptoms, varying from psychological distress to psychiatric disorders, as a result of the ongoing battle against many COVID-related adverse conditions [28].

The present study found a high prevalence of perceived stress among ICU staff. Since no previous studies had examined perceived stress among ICU staff in Qatar, we could not perform comparisons with previous works; however, our results are consistent with international studies [29, 30].

To the best of our knowledge, this is the first cross-sectional study assessing perceived stress and PTSD symptoms in ICU staff during the COVID-19 pandemic in Qatar and in the Middle East. In general, previous studies have shown that many HCWs experience psychological distress in widespread disasters because of their exposure, vulnerability, and long and intensive hours of work [31, 32]. Our results demonstrate that ICU staff in Qatar experienced high levels of perceived stress, and a probable diagnosis of PTSD was identified in a significant number of participants, and in a higher proportion of doctors than nurses. Interestingly, the current study found that the proportion of individuals experiencing moderate-to-severe perceived stress was lower among married than single respondents.

Although not statistically significant, variables such as occupation, previous ICU experience, and history of mental health disorders have demonstrated associations with a probable diagnosis of PTSD. Doctors were more likely to have a probable diagnosis of PTSD than nurses (adjusted OR 2.03; 95% CI 0.19–21.22; *p* = 0.556). In relation to this, nearly half of ICU and anesthetic staff included in a recent study conducted in England reported symptoms consistent with a probable diagnosis of PTSD, severe depression, anxiety, or problematic alcohol consumption [33].

In the current study, staff with previous ICU experience were less likely to have a probable diagnosis of PTSD than those without previous ICU experience (adjusted OR 0.41; 95% CI 0.11–1.62; *p* = 0.205). Respondents with a history of mental health concerns were more likely to have a probable diagnosis of PTSD than those without a history of mental health disorders (adjusted OR 2.26; 95% CI 0.17–30.11; *p* = 0.538).

Based on our findings, strategies that promote adaptive stress management and provide the skills needed to cope with stressful situations may reduce perceived stress and PTSD symptoms and improve the well-being of nurses and doctors working in the ICU during the COVID-19 pandemic.

### Limitations

Our cross-sectional study has some limitations. First, despite indicating observations and possible associations between variables, we could not infer causality. Second, the results were based on self-reported questionnaires designed to assess perceived stress and PTSD symptoms, which may differ from the clinical diagnostic criteria. Third, we were unable to discriminate pre-existing psychological distress from newly emerging symptoms. Fourth, although we targeted doctors and nurses, we did not explore specific job designations or years of experience, which could have influenced the results. Fifth, the impacts of secondary stressors (e.g., personal and relationship situations), which may have an effect on perceived stress among ICU staff, were not measured.

The estimated ORs in this study may not be sufficiently precise as the CIs are quite wide. This may be due to the small number of respondents in some subgroups. Larger scale studies are needed to confirm this association.

## Conclusions

In conclusion, perceived stress and other psychological outcomes, such as the development of PTSD symptoms, encountered by ICU staff during the COVID-19 pandemic may arise during subsequent waves of the virus or future health emergencies. It is important that interventions that encourage resilience in clinicians and mitigate this form of psychological distress are developed and implemented, a point that has been raised in previous studies [34]. Our current study demonstrates that HCWs’ response to outbreaks may have a range of psychological effects. Considering the likelihood of the long-term consequences that these may evoke and the possible association with reduced decision-making capacity, these mental health implications and perceived tensions become particularly alarming. The nature of the ICU position causes staff to be more vulnerable to critical situations that could adversely affect their mental health; thus, addressing this issue during epidemics and outbreaks is critical.

## Data Availability

All data related to this article is available with the first author upon reasonable request.
